# Chromosomal dynamics in *Senna*: comparative PLOP–FISH analysis of tandem repeats and flow cytometric nuclear genome size estimations

**DOI:** 10.3389/fpls.2023.1288220

**Published:** 2023-12-14

**Authors:** Thi Hong Nguyen, Byung Yong Kang, Hyun Hee Kim

**Affiliations:** Chromosome Research Institute, Department of Chemistry & Life Science, Sahmyook University, Seoul, Republic of Korea

**Keywords:** chromosomal rearrangement, FISH, genome dynamic, *Senna*, tandem repeat

## Abstract

**Introduction:**

Tandem repeats (TRs) occur abundantly in plant genomes. They play essential roles that affect genome organization and evolution by inducing or generating chromosomal rearrangements such as duplications, deletions, inversions, and translocations. These impact gene expression and chromosome structure and even contribute to the emergence of new species.

**Method:**

We investigated the effects of TRs on speciation in *Senna* genus by performing a comparative analysis using fluorescence *in situ* hybridization (FISH) with *S. tora*-specific TR probes. We examined the chromosomal distribution of these TRs and compared the genome sizes of seven *Senna* species (estimated using flow cytometry) to better understand their evolutionary relationships.

**Results:**

Two (StoTR03_159 and StoTR04_55) of the nine studied TRs were not detected in any of the seven *Senna* species, whereas the remaining seven were found in all or some species with patterns that were similar to or contrasted with those of *S. tora*. Of these studies species, only *S. angulata* showed significant genome rearrangements and dysploid karyotypes resembling those of *S. tora*. The genome sizes varied among these species and did not positively correlate with chromosome number. Notably, *S. angulata* had the fewest chromosomes (2*n* = 22) but a relatively large genome size.

**Discussion:**

These findings reveal the dynamics of TRs and provide a cytogenetic depiction of chromosomal rearrangements during speciation in *Senna*. To further elucidate the dynamics of repeat sequences in *Senna*, future studies must include related species and extensive repeatomic studies, including those on transposable elements.

## 1 Introduction

DNA tandem repeats (TRs) are abundant in heterochromatic regions within plant genomes, such as the (peri)centromere and (sub)telomere. These regions are hotspots of chromosomal rearrangement during genomic perturbations and may lead to the development of new species with altered chromosomal numbers or organization (Schubert and Lysak, 2011; Garrido-Ramos, 2017; Pellestor and Gatinois, 2018; Rosato et al., 2018; Hartley and O’neill, 2019). TRs and chromosomal rearrangements show close association because TRs both cause and result from chromosomal rearrangements and contribute to chromosomal evolution and speciation (Sousa and Renner, 2015; Louzada et al., 2020). The abundance and distribution of TRs vary among species, making them useful cytotaxonomic markers of phylogenetic relationships (Perumal et al., 2017). Additionally, chromosomal fragmentation or fusion may result in different basic chromosome numbers within a genus, which can result in dysploidy that could be ascending (species with more chromosomes) or descending (species with fewer chromosomes) (Mandáková et al., 2010; Schubert and Lysak, 2011; Mandáková et al., 2019). Tracking the distribution of these repeats across chromosomes and analyzing the dynamics of different repeat families may provide insights into the genomic history of closely related taxa (Perumal et al., 2017; Louzada et al., 2020).

The genus *Senna* Mill. (formerly *Cassia* L.) belongs to family Fabaceae and comprises approximately 350 species of herbs, shrubs, and trees that are extensively dispersed in tropical and subtropical regions (Azani et al., 2017; Bradley Morris et al., 2019). *Senna* species have been used for various economic and medicinal applications such as treating diabetes, microbial infections, skin diseases, gastrointestinal disorders, and inflammation (Oliur Rahman et al., 2013; Ongchai et al., 2019; Kang et al., 2020b). They contain a diverse range of important metabolites (alkaloids, anthraquinones, flavonoids, tannins, glycosides, steroids, terpenoids, and saponins) and display a wide range of *in vitro* and *in vivo* pharmacological activities (antidiabetic, anti-gonorrhea, antimicrobial, antioxidant, antipyretic, antinociceptive, antidepressant, and anti-inflammatory effects) (Puri, 2018; Kang et al., 2020a; Oladeji et al., 2021). However, the genus *Senna* presents challenges for cytogenetic studies owing to the diverse morphological and ecological characteristics of its members (Cordeiro and Felix, 2017; Nguyen et al., 2021; Ta et al., 2021; Waminal et al., 2021). Furthermore, *Senna* exhibits high levels of chromosomal variability with reported chromosome numbers ranging from 14–120 (Cordeiro and Felix, 2017). This variability complicates efforts to identify consistent patterns or relationships among *Senna* species and hampers the development of reliable cytogenetic markers for taxonomic and evolutionary studies.

Fluorescence *in situ* hybridization (FISH) is a powerful tool used in plant cytogenetic studies. It aids in the visualization of the DNA probes located on chromosomes within the nucleus, revealing information pertaining to chromosome structure, genome organization, and gene expression (Iovene et al., 2008; Waminal et al., 2018). FISH is particularly useful in plant breeding programs for investigating the evolutionary relationships between plant species and studying the impact of environmental stressors on chromosome structure and behavior (Sassi et al., 2019; Falistocco, 2020; de Moraes et al., 2021; Waminal et al., 2021).

Estimating genome size is essential for taxonomic purposes such as clarifying taxonomy and nomenclature, evaluating interspecific hybrid seedlings, and identifying ploidy levels (Bennett and Leitch, 2005). As genome size can be a distinguishing feature of individual species, genome size analysis assists in recognizing whole-genome duplication (WGD) and other genomic evolution events (Doležel et al., 2007; Bourge et al., 2018). Flow cytometry is now the preferred technique for genome size measurement (Bourge et al., 2018). Compared with alternative techniques such as Feulgen densitometry or genome sequencing, flow cytometry offers advantages such as simplified sample preparation, high throughput, and ability to estimate genome size, nuclear replication state, ploidy, and endopolyploidy levels (Doležel et al., 2007).

Exploring the chromosomal distribution of TRs via the FISH technique and comparative genomic analysis across multiple *Senna* species is pivotal for addressing cytogenetic research challenges within this genus. Notably, *Senna tora* L. (Roxb) (syn. *Cassia tora* L.) emerges as a widespread and representative species within the genus, subject to comprehensive genome sequencing to enhance our understanding of its biological evolution and relationship with other *Senna* species (Puri, 2018; Kang et al., 2020a; Waminal et al., 2021). Moreover, TRs are discerned to actively mediate the significantly rearranged descending dysploid karyotype of *S. tora*. Consequently, this study engaged in a comparative analysis, employing FISH alongside *S. tora* -specific pre-labeled oligo probes (PLOPs) and compared the genome sizes of several related *Senna* species. These selected species were *S. angulata* (Vogel) H. S. Irwin & Barneby, *S. artemisioides* subsp. *petiolaris* (DC.) Randell, *S. artemisioides* nothosubsp. *sturtii* (R.Br.) Randell, *S. hirsuta* var. *hirta* (Benth.) H. S. Irwin & Barneby, *S. lindheimeriana* (Scheele), *S. pallida* (Vahl) H. S. Irwin & Barneby, and *S. sophera* (L.) Roxb. which included diploid, dysploid, and polyploid karyotypes, provide a better understanding of whether the repeats identified are specific to *S. tora* or are conserved within the genus and scrutinize the impact of TRs on the speciation process within the *Senna* genus. Our ambition was to amass data and potentially yield cytogenetic evidence for historical, extensive chromosomal rearrangements and to enable a deeper comprehension of the evolutionary kinships among the species.

## 2 Materials and methods

### 2.1 Plant materials

Germinated seeds belonging to seven *Senna* species (mentioned in **Tables 1**, **2**) were provided by the National Plant Germplasm System (USDA, USA) and Rare Palm Seeds (Germany). The root tips were collected and treated with two mM 8-hydroxyquinoline at 18°C for 5 h to arrest cell growth at metaphase. The roots were fixed in Carnoy’s solution and stored in 70% ethanol until needed for chromosomal preparation.

**Table 1:** Chromosomal distribution of major *S. tora* tandem repeats in *Senna* species.

No.	Species	Accession number	Source	2*n*	5S rDNA	45S rDNA	Telomeric	StoTR01_86	StoTR03_178	StoTR04_55	StoTR05_180	StoTR06_159	StoIGS_463	Reference
1	*S. angulata* (Vogel) H. S. Irwin & Barneby	DLEG910253	USDA	22	pCen (3, 9)	NOR (9S) pCen (all)	Tel (all) pCen (1-3, 5-6, 8-11)	NOR (9S)	NOR (9S) pCen (1-8, 10, 11)	–	pCen (1,2, 4-8, 10, 11)	–	–	This study
2	*S. artemisioides* subsp. *petiolaris* (DC.) Randell	DLEG910206	USDA	56	IR (13S)	NOR (6S, 7S, 11S)	Tel (all)	NOR (6S, 7S, 11S)	–	–	–	–	–	This study
3	*S. artemisioides* nothosubsp. *sturtii* (R.Br.) Randell	DLEG920059	USDA	56	IR (12S)	NOR (3S, 4S, 5S)	Tel (all)	NOR (3S, 4S, 5S)	–	–	–	–	–	This study
4	*S. hirsuta* var. *hirta* (Benth.) H.S.Irwin & Barneby	PI322325 01	RPS	28	sTel (13S)	NOR (6S)	Tel (all) IRs (2S, 3S, 5L)	sTel (13S) NOR (6S)	sTel (1-14)	–	–	–	–	This study
5	*S. lindheimeriana* (Scheele)	DLEG910518	USDA	28	IR (14S)	NOR (11S)	Tel (all)	NOR (11S)	–	–	–	–	–	This study
6	*S. pallida* (Vahl) H. S. Irwin & Barneby	DLEG920148	USDA	28	pCen (13)	NOR (2S, 5S)	Tel (all) IR (7S)	–	–	–	–	–	–	This study
7	*S. sophera* (L.) Roxb.	DLEG900003	USDA	28	pCen (8)	NOR (7S)	Tel (all) IRs (1S, 2S, 4S)	pCen (8) NOR (7S)	–	–	–	–	pCen (8)	This study
8	*S. alata*	–	–	28	IR (13S)	NOR (2S, 7S, 11S)	Tel (all)	NOR (2S, 7S, 11S)	–	–	sTel (all)	NOR (2S, 7S, 11S)	sTel (1-6, 8, 10, 12-14) IRs (1-12, 14)	Ta et al. (2021)
9	*S. candolleana*	–	–	28	IR (13S)	NOR (4S, 11S)	Tel (all) IRs (1L, 7L)	NOR (4S, 11S)	–	–	sTel (all) IRs (1L, 7L)	–	sTel (1-3, 5-8, 10, 12-14) IRs (all)	Ta et al. (2021)
10	*S. corymbosa*	–	–	28	IR (13S)	NOR (11S)	Tel (all) IRs (1S, 2S, 3S)	NOR (11S)	–	–	sTel (all) IRs (1S, 2S, 3S)	NOR (11S)	sTel (5-10, 12-14) IRs (1-3, 5, 6, 8, 11, 13)	Ta et al. (2021)
11	*S. didymobotrya*	–	–	28	IR (9L)	NOR (1S, 2S, 4S, 11S)	Tel (all)	NOR (1S, 2S, 4S, 11S)	–	–	sTel (all)	–	–	Ta et al. (2021)
12	*S. floribunda*	–	–	28	IR (14L)	NOR (13S)	Tel (all) IRs (6S, 11S, 13S)	NOR (13S)	–	–	sTel (all) IRs (6S, 11S, 13S)	NOR (13S)	sTel (1-3, 5-14) IRs (2, 4-14)	Ta et al. (2021)
13	*S. occidentalis*	–	–	28	IR (13L)	NOR (2S)	Tel (all) IRs (1S, 3S, 4S)	NOR (2S)	–	–	sTel (all) IRs (1S, 3S, 4S)	–	–	Ta et al. (2021)
14	*S. multiglandulosa*	–	–	28	IR (13S)	NOR (5S)	Tel (all) IRs (10L, 11L)	NOR (5S)	–	–	sTel (all) IRs (10L, 11L)	NOR (5S)	–	Ta et al. (2021)
15	*S. sulfurea*	–	–	28	IR (13S)	NOR (2S, 3S, 7S)	Tel (all)	NOR (2S, 3S, 7S)	–	–	pCen (all)	–	–	Ta et al. (2021)
16	*S. siamea*	–	–	28	IR (4S)	NOR (2S, 5S, 6S)	Tel (all)	–	–	–	sTel (all)	–	–	Ta et al. (2021)
17	*S. tora*	–	–	26	IR (12S)	NOR (2S)	Tel (all) pCen (all) IRs (1L, 2S, 3S, 5S, 6-8L, 10L, 13L)	**Major:** paCen (1L, 3-5S, 6-8L, 10L, 13L) **Minor** pCen (11) paCen (4L, 9S, 12L)	**Major:** Cen (all) **Minor** IR (7L)	**Major:** pCen (1-8, 10-13) **Minor** pCen (9) IR (1L)	**Major:** Cen (1-3, 5-13) IR (6-8L) **Minor** Cen (4) IR (1L, 13L)	**Major:** NOR (2S) **Minor** IR (3S)	NOR (2S)	Waminal et al. (2021)

NOR, Nucleolar organizer region; Cen, centromeric; paCen, paracentromeric; pCen, pericentromeric; ITR, interstitial telomeric region; IR, interstitial region; Tel, telomeric region; sTel, subtelomeric region; S, short arm; L, long arm; Numbers in parentheses represent the chromosome number with FISH signals for each corresponding repeat; USDA, National Plant Germplasm System (USA); RPS, Rare Palm Seeds (Germany).

**Table 2:** Genome size of seven *Senna* species.

No.	Species	Chromosome number (2*n*)	DNA content/2C (pg)	DNA content/1C (pg)	Genome size/1C (Mbp)
1	*S. angulata*	22	2.35	1.18	1150.57
2	*S. artemisioides* subsp. *petiolaris*	56	2.15	1.08	1053.79
3	*S. artemisioides* nothosubsp. *sturtii*	56	2.43	1.22	1190.07
4	*S. lindheimeriana*	28	1.50	0.75	732.24
5	*S. hirsuta* var. *hirta*	28	1.29	0.65	631.27
6	*S. pallida*	28	2.18	1.09	1064.16
7	*S. sophera*	28	1.57	0.78	767.05

### 2.2 Chromosome spread preparation

Sporophytic metaphase chromosome slides was prepared based on the method described by Waminal and Kim (2012) with some modifications. Briefly, small pieces of the meristematic tips (approximately 2 mm) were treated with a pectolytic enzyme solution containing 2% Cellulase RS (Phytotechnology Laboratories, Lenexa, KS, USA) and 1% Pectolyase Y-23 (Duchefa, Haarlem, The Netherlands) in a 100 mM citrate buffer for 2 h at 37°C. After washing with distilled water, the roots were placed in chilled Carnoy’s solution and vortexed for 30 s at room temperature. The pellet obtained was suspended in an acetoethanol mixture, and the cellular suspension was pipetted onto glass slides that had been prewarmed in a humid chamber. The slides were air-dried, fixed in 2% formaldehyde (Merck Schuchardt OHG, Hohenbrunn, Germany) for 5 min, dipped in distilled water, and dehydrated using increasing concentrations of ethanol (70%, 90%, and 100%).

### 2.3 Repeat mining, probes preparation, and fluorescence *in situ* hybridization

The paired-end reads of the genome of *S. tora* were obtained and used for quality trimming, read sampling, and repeat clustering. *S. tora*-specific TRs were identified in our previous study (Waminal et al., 2021) using low-coverage sequences and short-read clustering with TAREAN (Novák et al., 2017). All the prelabeled oligonucleotide probes (PLOPs) used for FISH analysis in this study are listed in **Table 3**.

**Table 3:** List of pre-labeled oligonucleotide probes (PLOPs) used in this study (Waminal et al., 2021).

Name	Oligo name	PLOP sequences (5′–3′)	Length (bp)	Modification
StoTR01_86	StoTR01_86_OP1	TTAATCAGTTTTCGCCGATGAGTGTTTCG	29	5′-FAM
	StoTR01_86_OP2	CATCAGTTTTCGCCAATGAGTGTTTCG	27	
Sto_Tel	Tel_UniOP_*Arabidopsis*	TTTAGGGTTTAGGGTTTAGGGTTTAGGGT	29	ATTO425
StoTR03_178	StoTR03_178_OP1	CCGGAATATGTTAAGACATGATCCACGCT	29	5′-Cy5
	StoTR03_178_OP2	ATCTCAGAAACCTTCACGAATTACGAGGC	29	
	StoTR03_178_OP3	CCGGAGTGGTTTTGATGCTCCAATTGGA	28	
StoTR04_55	StoTR04_55_OP	GCGAAAACTGATTAAAAAAAGAAAAATGAATATCAAG	37	5′-AMCA
StoTR05_180	StoTR05_180_OP1	GATTTAATGCTCGAATGGGGCTCGTGATC	29	5′-Texas Red
	StoTR05_180_OP2	GTTGTTGCACAAGTGAGTCAAACCGATC	28	
	StoTR05_180_OP3	TGTTTAGACATGACTTGACACACCTTCCA	29	
	StoTR05_180_OP4	TGAGTTCTTTTGAGATTCAATCGCGATTT	29	
StoTR06_159	StoTR06_159_OP1	TGCATATGCTGGGTCAAAATGAAGCCTAT	29	5′-Cy3
	StoTR06_159_OP2	AGGCTTCCTTGTGTCATAGGCTTCATTTT	29	
StoIGS_463	StoIGS_463_PLOP1	AAACCAATATATATTCTATTTTTCGTGATT	30	5′-FAM
	StoIGS_463_PLOP2	CAAATGATTGATAAGCCTTTAATTTTATTA	30	
	StoIGS_463_PLOP3	GAAATTTTGGGGTTAAGCTTATATATTTTT	30	
Sto_45S	18SrDNA_UniOP_1	CCGGAGAGGGAGCCTGAGAAACGGCTAC	28	5′-Cy3
	18SrDNA_UniOP_2	ATCCAAGGAAGGCAGCAGGCGCGCAA	26	
	18SrDNA_UniOP_3	GGGCAAGTCTGGTGCCAGCAGCCGCGGT	28	
	18SrDNA_UniOP_4	TCGAAGACGATYAGATACCGTCSTAGT	27	
	18SrDNA_UniOP_5	CTGAAACTTAAAGGAATTGACGGAAGG	27	
	18SrDNA_UniOP_6	GGAGCCTGCGGCTTAATTTGACTCAAC	27	
	18SrDNA_UniOP_7	GGTGGTGCATGGCCGTTCTTAGTTGGTGG	29	
	18SrDNA_UniOP_8	ACGTCCCTGCCCTTTGTACACACCGCCCGTC	31	
	5.8SrDNA_UniOP_1	AAYGACTCTCGGCAACGGATATCTMG	26	
	5.8SrDNA_UniOP_2	CWYGCATCGATGAAGAACGTAGCRA	25	
	5.8SrDNA_UniOP_3	GCGATACTTGGTGTGAATTGCAGAATC	27	
	5.8SrDNA_UniOP_4	GTGAACCATCGAGTYTTTGAACGCAAGT	28	
Sto_5S	5SrDNA_ang_1	GGATGCGATCATACCAGCACTAAAGCACCG	30	5′-Alexa Fluor 488
	5SrDNA_gym_1	GRGTGCGATMATACCASCGYTWRYGYA	27	
	5SrDNA_cranial_1	GYYTAYRGCCAYACCACCCTGRRHRCG	27	
	5SrDNA_ang_2	CCCATCAGAACTCCGAAGTTAAGCGTGCT	29	
	5SrDNA_gym_2	ATCCSATCAGAACTCCGYARTTAAGCR	27	
	5SrDNA_cranial_2	GATCTCGTCYGATCTCGGAAGCTAAGC	27	
	5SrDNA_ang_3	GCGAGAGTAGTACTAGGATGGGTG	24	
	5SrDNA_gym_3	TTGGGYYRGAGTAGTACTRGGATGGGT	27	
	5SrDNA_cranial_3	GTCGGGCCYGGTYAGTACTTGGATGGG	27	
	5SrDNA_ang_4	CCTGGGAAGTMCTCGTGTTGCAYYCC	26	
	5SrDNA_gym_4	CTCYYGGGAAGTCCYRRTRTYGCACCC	27	
	5SrDNA_cranial_4	CYGCCTGGGAATACCRGGTGYYGTARG	27	

A 40 µL hybridization mixture was prepared by adding 100% formamide, 50% dextran sulfate, 20× SSC, 50 ng/µL of each probe, and Sigma water, followed by denaturation at 80°C and incubation at 37°C overnight. This hybridization mixture was used to perform FISH. The slides prepared earlier were washed with 2× SSC at 22-25°C (RT) and dehydrated consecutively in 70%, 90%, and 100% ethanol for 3 min each time. Finally, they were counterstained with DAPI-Vectashield (Vector Laboratories, Newark, CA, USA) and visualized with an Olympus BX53 (Olympus, Tokyo, Japan) fluorescence microscope system equipped with a Leica DFC365 FS CCD camera (Leica Microsystems, Wetzlar, Germany). The images were captured using the Cytovision software (Leica Microsystems) and enhanced using Adobe Photoshop CS6 (Adobe Inc., San Jose, California, USA). The chromosomes were analyzed using ImageJ 1.2 software and paired based on FISH signals, chromosome length, centromere position and morphological characteristics. Chromosome typing was performed as described previously by Levan et al. (1964).

### 2.4 Genome size measurement and assessment of nuclear DNA content using flow cytometry

The total nuclear DNA content (2C-DNA value) of the seven *Senna* species were measured using flow cytometry by following the method outlined by Bourge et al. (2018). Briefly, fresh leaves were collected from ten individuals of each species and mixed with an internal reference standard (*Dendropanax morbifera*, 2C DNA *=* 4.09 pg). In a petri dish, the samples were treated with the isolation LB01 buffer and passed through two nylon mesh filters (CellTrics Filters, Sysmex Asia Pacific, Singapore) with pore sizes of 50 µm and 20 µm, respectively. The filtered nuclei were stained with propidium iodide (Sigma-Aldrich, St. Louis, MO, USA, cat. no. P4170; Molecular Probes; cat. no. P3566) and RNase A (Sigma-Aldrich, St. Louis, MO, USA, cat. no. R5000) and analyzed using a CytoFLEX flow cytometer (Beckman Coulter, California, USA). The DNA content of the samples was computed by analyzing the peaks of the standards and samples using CytoExpert v2.3 software (Beckman Coulter Inc., Pasadena, CA, USA). The 2C values were calculated by estimating the linear fluorescence intensity of the stained nuclei for each species and the internal standard. The relative genome size was determined using a formula following Nguyen et al. (2023). The coefficient of variation (CV) was below 3% on average and did not exceed 7%.

## 3 Results

### 3.1 Chromosome counts

Although 2*n* = 28 has been the predominantly reported number of chromosomes in most *Senna* species, variations in chromosome number occur due to polyploidy or disploidy, resulting in 2*n* = 56 (*S. artemisiodes* subsp. *petiolaris* and *S. artemisioides* nothosubsp. *sturtii*) or 2*n* = 22 (*S. angulata*) chromosomes, respectively. In this study, *S. angulata* exhibited a diploid chromosome count of 2*n* = 22, contrasting with prior findings of 2*n* = 26 reported by Biondo et al. (2005). Notably, this is not the inaugural documentation of chromosome numbers for *S. artemisiodes* or *S. hirsuta*. However, our research marks the maiden report concerning chromosome numbers for distinct variants of these species, encompassing *S. artemisiodes* subsp. *petiolaris* and *S. artemisioides* nothosubsp. *sturtii* (2*n* = 56), as well as *S. hirsuta* var. *hirta* (2*n* = 28). Meanwhile, *S. lindheimeriana*, *S. pallida* and *S. sophera* displayed a consistent chromosome count of 28, aligning with previously published data.

### 3.2 Distribution of nine *S. tora*-TR probes

**Figure 1:**
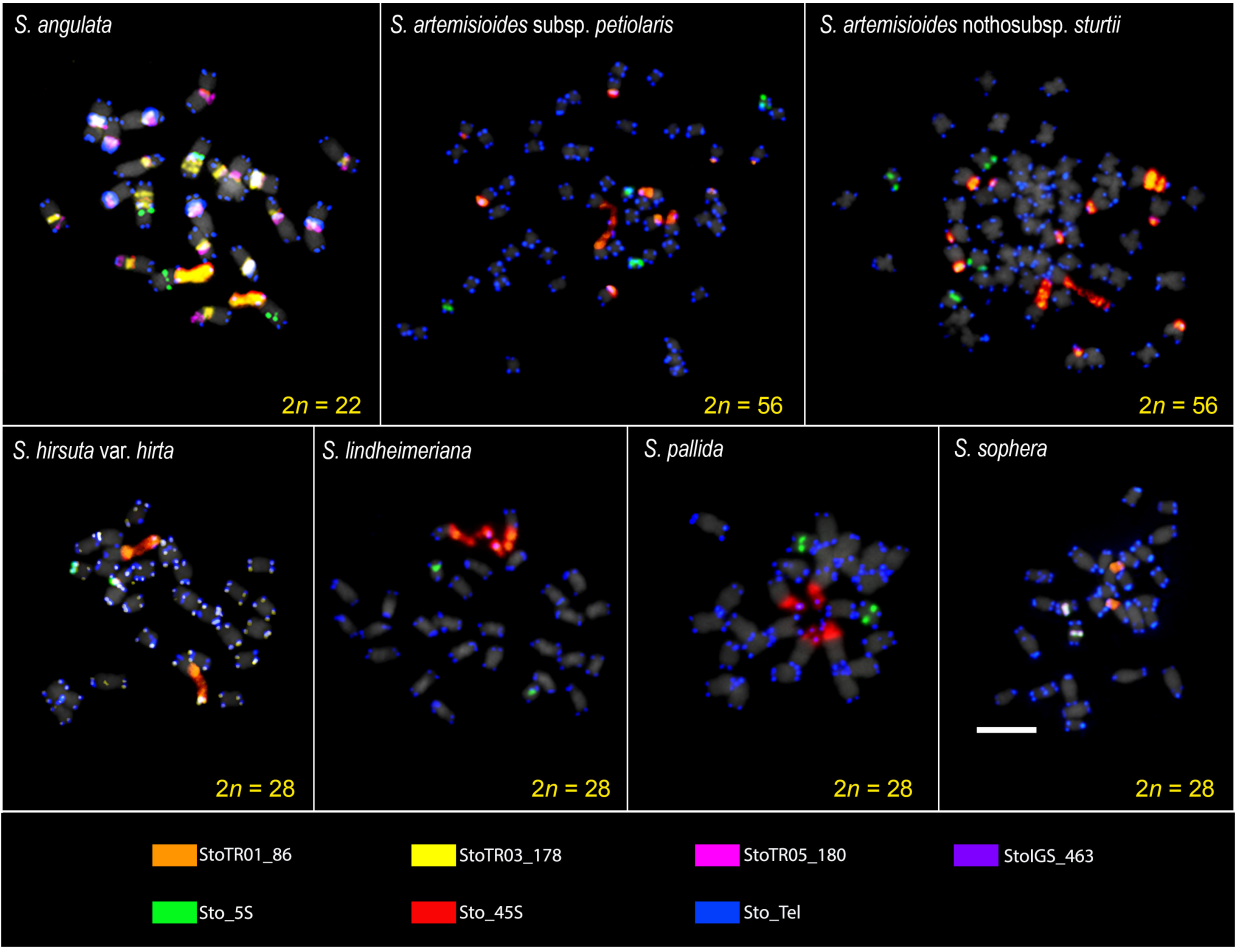
FISH images of *S. tora*-TRs on metaphase chromosomes of the seven *Senna* species. Seven TRs showed signals in all or some species with varied patterns. The signal patterns of individual probes are shown in **Supplementary Data** (**Figures 1S–5S**). Scale bar = 5 µm.

**Figure 2:**
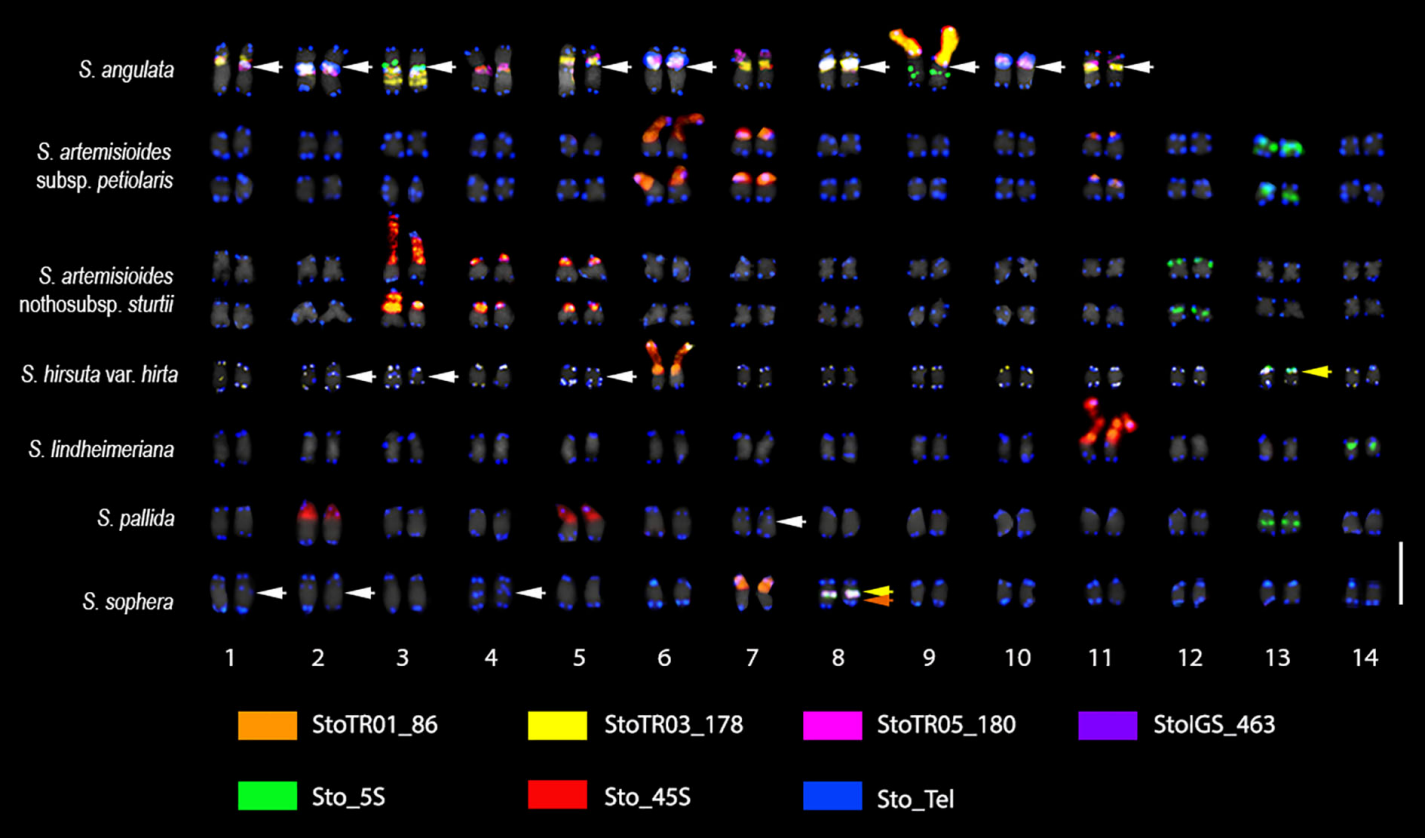
FISH karyogram of seven *Senna* species obtained using *S. tora*-TRs. StoTR01_86 is mainly colocalized at 45S rDNA sites in all species except in *S. pallida*, which did not carry the signal; *S. hirsuta* and *S. sophera* show an additional signal pair colocalized at the 5S rDNA region (yellow arrows). White arrows indicate ITRs in *S. angulata*, *S. hirsuta*, *S. pallida*, and *S. sophera*. The orange arrow shows StoIGS_463 signals colocalized with 5S rDNA in *S. sophera*. StoTR05_180 is present only in *S. angulata* at subcentromeric sites of nine chromosomes. StoTR03_178 is shown at the centromeric regions of *S. angulata* chromosomes; in contrast, they are observed at the subtelomeric sites in *S. hirsuta*. The karyogram of each species showing the individual TR distribution are shown in **Supplementary Data** (**Figures 6S–12S**). Scale bar = 5 µm.

FISH analysis of the nine TRs examined showed that two were not present in any of the seven *Senna* species (StoTR06_159 and StoTR04_55), whereas the other seven were detected in all or some of the species (**Table 1**). The 5S rDNA, 45S rDNA, and telomeric repeats were present in all species; StoTR01_86 was present in all species except *S. pallida*; and StoTR01_178 and StoTR06_180 were present in only two and one species, respectively. Furthermore, StoIGS_463 was present only in *S. sophera* (**Figures 1**, **2**; **Table 1**).

When examining seven different *Senna* species, the 45S rDNA loci exhibited higher diversity than the 5S rDNA. Except for *S. angulata*, all species were found to have a single pair of Sto_5S rDNA in the pericentromeric or interstitial regions of the short arms of their respective chromosomes. However, in the case of *S. hirsuta*, Sto_5S rDNA subtelomeric signals were observed. Sto_45S rDNA was detected in one, two, or three distinct loci in these species. *S. angulata* was unique in exhibiting two pairs of 5S rDNA, while all its chromosomes displayed 45S rDNA signals in the (peri)centromeric regions, in addition to one pair of NOR signals. All seven species demonstrated Sto_Tel signals at the terminal regions of all chromosomes. Beyond these canonical sites, interstitial telomeric repeat (ITR) signals were identified in *S. angulata*, *S. hirsuta*, *S. pallida*, and *S. sophera*. Specifically, *S. angulata* displayed ITR signals in the (sub)centromeric regions of nine chromosomes. Conversely, ITRs were observed in short arms of chromosomes 2 and 3 and long arm of chromosome 5 in *S. hirsuta*, the short arm of chromosome 7 in *S. pallida*, and the short arms of chromosomes 1, 2, and 4 in *S. sophera*.

The distribution of StoTR01_86 in the chromosomes of the seven *Senna* species was examined in the present study and revealed to be completely different from that observed in *S. tora*, which harbors StoTR01_86 in the pericentromeric regions of all chromosomes. However, for six species of *Senna* studied here, it was found colocalized with all 45S rDNA loci, and *S. pallida* did not show any signal for StoTR01_86. Notably, *S. hirsuta* and *S. sophera* displayed an extra pair of StoTR01_86, which was colocalized at the site of the 5S rDNA signals. These findings are illustrated in **Figures 1**, **2** and summarized in **Table 1**.

In *S. tora*, both StoTR03_178 and StoTR05_180 exhibited a centromeric distribution across all chromosomes. However, in *S. angulata*, StoTR03_178 signals were observed at the centromeres of all chromosomes except for two, one of which carried the 45S rDNA signal. In this species, StoTR03_178 was also detected at pericentromeric regions in all chromosomes except the one with the 45S rDNA signal, where it was found at the NOR sites. In contrast, *S. hirsuta* displayed StoTR05_180 signals that colocalize with Sto_tel at chromosome termini and some interstitial regions.

Finally, in *S. sophera*, we identified only one pair of StoIGS_463 signals colocalized with the 5S rDNA signal, whereas in *S. tora*, these signals were exclusively located at the NOR site.

### 3.3 Flow cytometric genome size estimation

**Figure 3:**
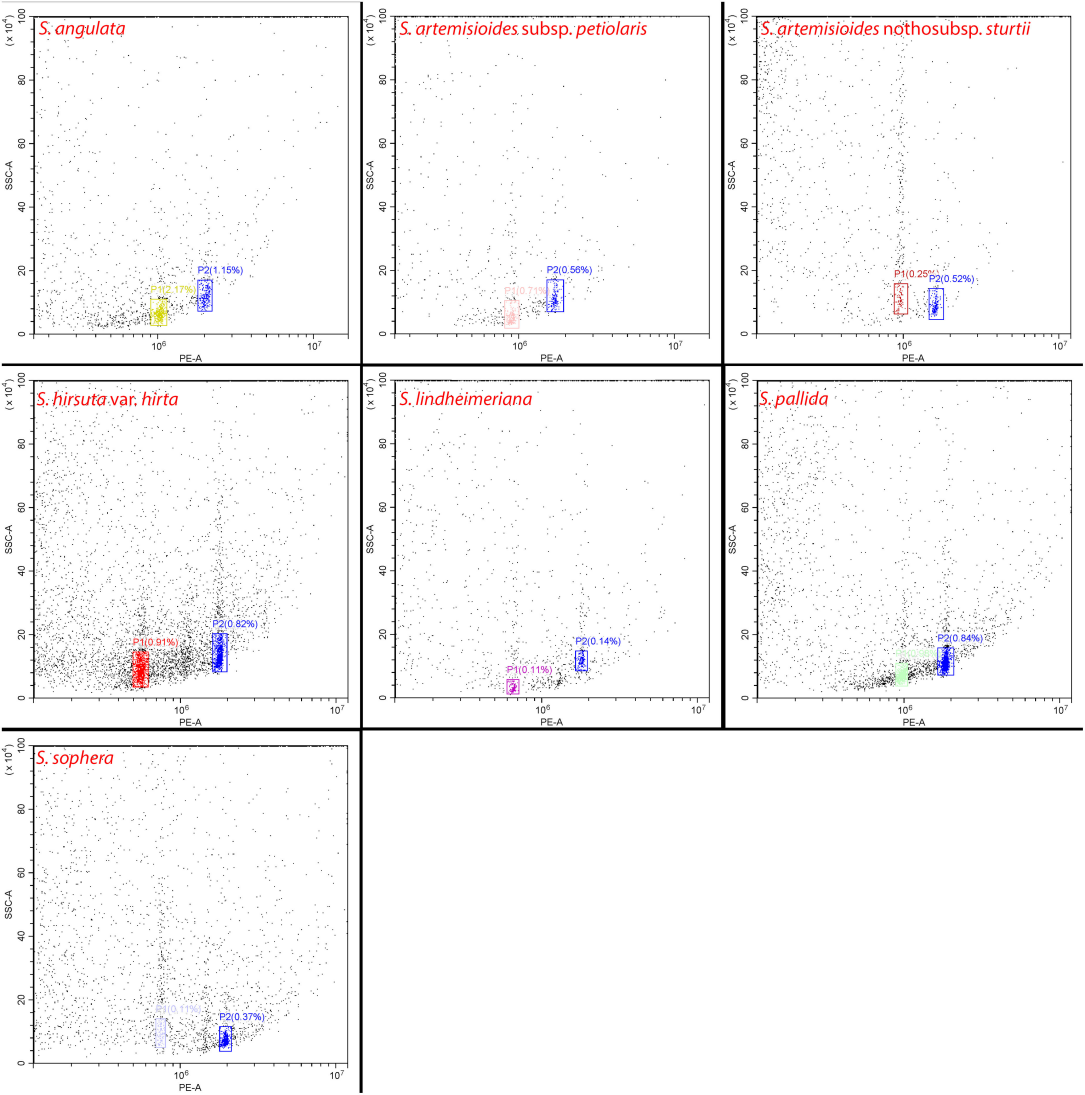
Flow cytometric dot plots of relative fluorescence intensities of propidium iodide-stained nuclei isolated from seven *Senna* species and internal standard *Dendropanax morbifera* (in blue, 2C = 4.09 pg).

Flow cytometric analysis of the genome size (2C-value) of all seven *Senna* species using *Dendropanax morbifera* (2C = 4.09 pg) as an internal standard produced histograms with well-defined peaks and low coefficients of variation (< 3.0%) (**Figure 3**). This supported the reliability of flow cytometric assessments. The genome size varied among the *Senna* species with values ranging from 2C = 1.29 pg (631.27 Mb) in *S. hirsuta* to 2C = 2.43 pg (1190.07 Mb) in *S. artemisioides* nothosubsp. *sturtii*. In fact, the DNA content varied even among the species with the same number of chromosomes, that is, 2*n* = 28 (*S. lindheimeriana*, *S. hirsuta*, *S. pallida*, and *S. sophera*) and 2*n* = 56 (*S. artemisioides* subsp. *petiolaris* and *S. artemisioides* nothosubsp. *sturtii*). Notably, *S. angulata*, which had the fewest chromosomes (2*n* = 22), has a relatively large genome size, ranking it second among the seven species ahead of *S. artemisioides* subsp. *petiolaris* (2*n* = 56) (**Figure 1**; **Table 2**).

## 4 Discussion

Karyotypic data play a crucial role in assessing genetic relationships, shedding light on the origin and divergence of species (Bhat and Wani, 2017). This information encompasses critical factors such as chromosome number, size, morphology, satellite DNA position, and chromosome banding patterns (Guerra, 2008). When comparing the findings to previous reports (Irwin and Turner, 1960; Biondo et al., 2005), it becomes evident that *S. angulata* exhibits varying chromosome counts. This discrepancy could stem from possible botanical misidentification, variations in sample accession numbers across different studies, or inherent genetic diversity within the species (Guerra, 2008). *Senna* species have traditionally been documented with a basic chromosome number of *x* = 14 being the most common (Chaulagain and Sakya, 2002; Resende et al., 2014), often considered a polyploid derivative of *x* = 7, a characteristic shared across all Caesalpinioideae (Elaine et al., 2005). Our analysis identified a chromosome number of 2*n* = 28 in four of the seven *Senna* species examined. However, *S. angulata* showed a chromosome number of 2*n* = 22. This discrepancy may be attributed to dysploidization events, such as chromosome fusion, occurring during *Senna* evolution (Elaine et al., 2005; Ferreira et al., 2010), also observed in *S. tora* (Waminal et al., 2021). It’s important to note that interspecies chromosome variation within a genus is not unique to *Senna* and has been observed in other genera like *Brassica*, *Cucumis*, *Nothoscordum*, and *Brachyscome* (Maluszynska and Heslop-Harrison 1993; Watanabe et al., 1995; Koo et al., 2010). Dysploidization, as seen in this case, is a recognized driver for speciation (Freyman and Höhna, 2018). Furthermore, our study unveiled a chromosome number of 2*n* = 56 in *S. artemisioides* subsp. *petiolaris* and *S. artemisioides* nothosubsp. *sturtii*. Such an observation suggests that polyploidy events may play a role in the speciation of these particular *Senna* subspecies.

The 45S rDNA repeats show complex distribution and organization patterns in plant genomes with wide variation between species (Roa and Guerra, 2012). Previous studies have investigated this phenomenon by analyzing the localization patterns on chromosomes in several plant species such as *Arabidopsis thaliana*, *Brassica rapa*, *Cucumis* species, *Lilium distichum*, and *Solanum chacoense* (Fukui et al., 1998; Hwang et al., 2015; Zhang et al., 2016). The mechanisms underlying rDNA distribution and homogenization are diverse and include transposition, unequal crossing-over, and gene conversion. In the present study, *S. angulata* presented 45S rDNA signals in both the NOR and (peri)centromeric regions of the chromosomes, suggesting that multiple clusters of rDNA repeats were scattered throughout the genome. As a tandemly repeating locus, rDNA is vulnerable to copy-number mutations, especially when exposed to topological stress resulting from the unwinding of the helical DNA structure and collisions between replication and transcription machinery due to frequent transcription activities (Salim and Gerton, 2019). Laboratory studies have consistently shown that exposure to various stressors can induce rapid changes in rDNA copy numbers (Kobayashi, 2011; Paredes et al., 2011; Aldrich and Maggert, 2015; Jack et al., 2015; Salim et al., 2017), highlighting the environmental sensitivity of the rDNA locus (Salim and Gerton, 2019). Furthermore, rDNA content is intimately connected to genome stability and susceptibility to stress (Kobayashi and Sasaki, 2017). This relationship may elucidate the expansion of 45S rDNA copies in all chromosomes of *S. angulata*, which is a dysploid karyotype that may have experienced numerous rearrangements during speciation, adaptation, survival, and evolution. The homogenization of these repeats may have occurred over time through mechanisms similar to those observed in other plant species, like *Atropa belladonna, Thinopyrum intermedium* (Mahelka et al., 2013; Volkov et al., 2017). In fact, transposition, unequal crossover events, and homogenization through gene conversion are possible mechanisms that may explain the observed localization and distribution of rDNA repeats in *S. angulata*. The presence of NOR signals on multiple chromosomes in *S. artemisioides* subsp. *petiolaris* and *S. artemisioides* notosubsp. *sturtii* suggest that they may have undergone chromosomal rearrangements, resulting in the duplication of the NOR regions. Further research is required to determine the mechanisms underlying the localization and distribution of rDNA repeats in these plant species.

The presence of StoTR01_86 at the 45S rDNA locus in most *Senna* species studied, except *S. pallida*, suggests its involvement in chromosomal rearrangements following the divergence of *Senna* species from their common ancestor, with the karyotype represented by *S. pallida*. This process may have led to the correction of error StoTR01_86 which was also observed in *S. siamea* in the study by Ta et al. (2021) (**Table 1**). Subsequently, specific chromosomal rearrangements in *S. tora* may have caused the recent movement of StoTR01_86 from the IGS region to the pericentric region of all chromosomes (Waminal et al., 2021). In addition, this study also revealed that StoTR01_86 had undergone expansion, relocating from IGS region of the 45S rDNA to the 5S rDNA in *S. hirsuta* and *S. sophera*. The underlying mechanism governing this expansion and movement of the repetitive DNA element StoTR01_86 likely involves transpositional events, which are molecular processes enabling specific DNA sequences, including repetitive elements, to mobilize within a genome. These findings underscore the dynamic nature of plant genomes, the potential influence of repetitive elements on genome evolution, and the intriguing role of StoTR01_86 in shaping the genetic landscape of *Senna* species.

In our previous investigation, Ta et al. (2021) reported the co-localization of StoTR05_180 with Sto_Tel at subtelomeric regions in most nine studied species, except for *S. sulfurea*, which exhibited pericentromeric signals across all chromosomes, a similar pattern in *S. tora*, as detailed in **Table 1**. Our current study also detected StoTR05_180 in the pericentromeric region of nine chromosomes in *S. angulata*. This intriguing pattern suggests that the transposition of these TR loci from subtelomeric to pericentromeric regions may have occurred across multiple species, including *S. angulata*, *S. sulfurea*, and *S. tora*. We propose that this transposition event may have involved the ancestral karyotypes of these species. However, it is also plausible that this transposition event occurred through relatively random chromosomal rearrangement events. One potential mechanism for such concerted transposition of tandem repeat arrays is chromoplexy, a complex process involving extensive chromosomal rearrangements across multiple chromosomes, as previously described (Comai and Tan, 2019; Pellestor and Gatinois, 2020). Notably, microhomologies between telomeric and pericentromeric regions make these regions susceptible to chromosomal inversions (He et al., 2013).

It is noteworthy that certain evolutionary events have occurred in the case of *S. angulata*, *S. sulfurea*, and *S. tora*. Specifically, these species have experienced the loss of subtelomeric StoTR05_180 loci while concurrently amplifying pericentromeric loci. In the case of *S. tora*, an intriguing development has occurred wherein a novel centromeric repeat, StoTR03_178, has emerged. It is highly plausible that StoTR03_178 may have evolved from StoTR05_180 (Waminal et al., 2021). The disruption of the epigenetic landscape immediately following chromosomal rearrangements appears to have facilitated the transformation of StoTR05_180 into a novel centromeric repeat. In *S. tora*, this transformation may have influenced the positioning and stabilization of StoTR03_178 variants as centromeric elements. The abundance of StoTR03_178 copies relative to StoTR05_180, coupled with its centromeric localization, indicated a notable shift in centromeric repeat preference towards StoTR03_178. This shift likely resulted from restoring proper meiotic pairing following genomic perturbations (Ma and Gustafson, 2005; Schubert and Vu, 2016). Notably, in this study, similar dynamics were also observed in *S. angulata*. This may support the idea that *S. angulata* and *S. tora* underwent comparable evolutionary events involving StoTR03_178 and StoTR05_180 during the development of dysploid karyotypes.

Subrepeat elements located within the IGS play a crucial role in shaping the dynamics of both the IGS itself and the 45S rDNA, thereby influencing genomic variability (Jo et al., 2011; Mirete et al., 2013; Havlová et al., 2016; Ma et al., 2020). The rearrangement and excision of IGS repeat elements introduce variability in IGS length across species, even within the same taxon (Krawczyk et al., 2017). Waminal et al. (2021) previously reported that StoIGS_463 is a duplicated 463 bp sequence and two StoIGS_463 copies were located in the intergenic spacer (IGS) region of *S. tora* 45S rDNA. However, our recent research revealed that StoIGS_463 was found exclusively in *S. sophera* but adjacent to 5S rDNA, not 45S rDNA, as observed in *S. tora*. Intriguingly, StoIGS_463 was also detected in subtelomeric or interstitial chromosomal regions of several other species, including *S. alata*, *S. candolleana*, *S. corymbosa*, and *S. floribunda* (Ta et al., 2021). This finding suggests that the IGS segment has undergone relocation and amplification within alternative chromosomal regions in these species, departing from its original position at the NOR site, as observed in *S. tora* (Waminal et al., 2021). These IGS transposable elements exhibit characteristics like transposons, migrating in and out of the 45S rDNA IGS and experiencing amplification within diverse chromosomal locations (Almeida et al., 2012; Waminal et al., 2021). However, the precise mechanisms governing this translocation remain unclear. Combining with our previous research, we propose that the 45S rDNA IGS may act as a carrier during chromosomal rearrangements, occasionally anchoring transposed TR fragments within a taxonomic group and signify the recent involvement of IGS in chromosomal rearrangements (Ta et al., 2021).

Telomeric repeats are typically found at the ends of chromosomes in most eukaryotes. They play an essential role in preventing chromosomal damage (Fuchs et al., 1995; Muraki et al., 2012). Nevertheless, interstitial telomeric repeats (ITRs) are also known to occur in some animal and plant species; however, their size, number, and distribution vary within and between species (He et al., 2013; Sousa and Renner, 2015; Souza et al., 2016; Nguyen et al., 2021). In this study, four of the seven *Senna* species, namely, *S. angulata*, *S. hirsuta*, *S. pallida*, and *S. sophera* showed ITR signals and chromosomal rearrangements involving Sto_Tel. This suggests that these species shared a common ancestor that underwent these rearrangements. The absence of ITR signals in the other species may be attributed to the repeat loci being fixed and reduced, making them challenging to detect. ITRs in plants are considered cytological landmarks of chromosomal rearrangements, which could result from various mechanisms, such as ancestral chromosomal fusion events, translocation, inversion, and equivocal dispersion of telomeric DNA (Murat et al., 2010; Sousa and Renner, 2015; Wang et al., 2015; Rosato et al., 2018; Maravilla et al., 2021). ITRs in the (peri)centromeric regions may be remnants of past end-to-end fusion between non-homologous chromosomes, leading to descending dysploidy in *S. angulata*. Telomeric sequences may also be amplified through mechanisms such as those involved in the genomic evolutionary dynamics of satellite DNA (Charlesworth et al., 1994; Cohen et al., 2008; He et al., 2013; Sousa et al., 2014). Moreover, the DNA double-strand break repair mechanisms may also contribute to the presence of short stretches of ITRs (Lin and Yan, 2008). Furthermore, the mechanisms mentioned are not mutually exclusive and may act together in the evolutionary turnover of plant ITRs.

The observed data indicated that genome size was not directly affected by the number of chromosomes across the examined species. This might underscore the multifaceted nature of genome organization, influenced by factors such as gene density, non-coding regions, and evolutionary events like chromosome fusions and fissions. Notably, species with fewer chromosomes, such as *S. angulata*, can have a larger genome size attributed to more giant chromosomes or more extensive non-coding regions. Similarly, species like *S. artemisioides* nothosubsp. *sturtii*, despite having more chromosomes, exhibit a genome size comparable to those with fewer chromosomes. The abundant amount of TRs in *S. angulata* may be the reason for the larger genome size of this species compared to other studied species. These observations highlight the importance of delving deeper into the genomic architecture rather than relying solely on chromosome number or genome size as proxies for genetic complexity or evolutionary adaptation.

Finally, when examining the studied TRs, it becomes evident that StoTR01_86 stands out as a relatively conserved marker in most *Senna* species. In contrast, StoTR04_55 exhibits species specificity, exclusively manifesting a distinct signal within *S. tora*. The remaining TRs display varying degrees of presence and expression patterns among different species, differing from those observed in *S. tora*. These findings vividly illustrate the dynamic role of TRs in the speciation and evolutionary processes within the *Senna* genus. Remarkably, TRs appear more abundant in species with dysploid karyotypes, such as *S. angulata* and *S. tora*, with signals concentrated predominantly in (peri)centromeric regions, which serve as hotpots for chromosomal rearrangements (Schubert and Lysak, 2011; Pellestor and Gatinois, 2018; Rosato et al., 2018; Hartley and O’neill, 2019). Interchromosomal rearrangements, encompassing arm translocations, end-to-end translocations, and nested chromosome insertions, could give rise to dysploid species with reduced chromosome numbers within these lineages (Rousselet et al., 2000; Mandáková and Lysak, 2018). Consequently, these observations further substantiate the critical role of TRs as drivers of dysploidy-mediated karyotype evolution and the speciation process.

## 5 Conclusion

In conclusion, TRs are essential for plant genomes, and they affect genome organization and evolution. We observed a combination of shared and independent evolutionary patterns across the studied species, revealing the dynamics of the TRs and providing a better understanding of the evolutionary relationships among these species. Additionally, this study reveals the dynamics of *Senna* TRs during speciation. Furthermore, these data offer a cytogenetic perspective on the expansion, contraction, and rearrangement of repeat families within the repeat repository of a lineage. Further studies are required to comprehensively investigate the dynamics of the repeats within the *Senna* genus. Future studies should encompass the creation of additional TR elements and their applications in a broader array of related species.

## Data availability statement

The original contributions presented in the study are publicly available. This data can be found here: NCBI, GCA_014851425.1.

## Author contributions

TN: Data curation, Formal analysis, Investigation, Methodology, Software, Visualization, Writing – original draft, Writing – review & editing. BK: Investigation, Writing – review & editing. HK: Conceptualization, Funding acquisition, Project administration, Supervision, Validation, Writing – review & editing.
