# Supplementary material for: Chromosomal dynamics in Senna: comparative PLOP–FISH analysis of tandem repeats and flow cytometric nuclear genome size estimations
Source: Front Plant Sci. 2023 Dec 14;14:1288220. doi: 10.3389/fpls.2023.1288220 (PMC10762312; doi:10.3389/fpls.2023.1288220)
Supplement: Supplementary file 1 [file DataSheet_1.pdf]

Supplementary Material

1.1 Supplementary Figures

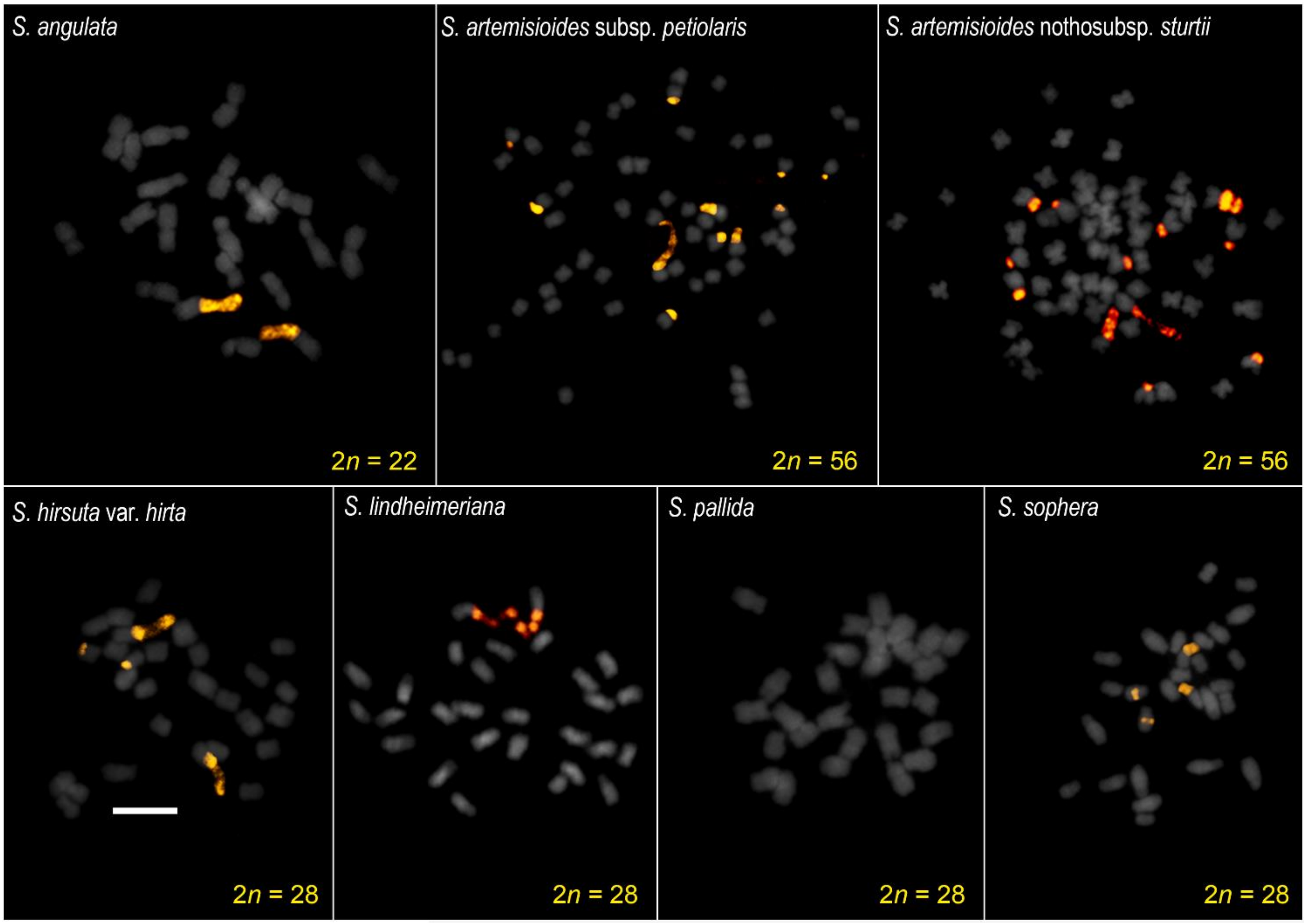

Figure 1S. Distribution of StoTR01_86 in metaphase spreads in seven *Senna* species. StoTR01_86 signals are labeled orange. Scale bar = 5 µm

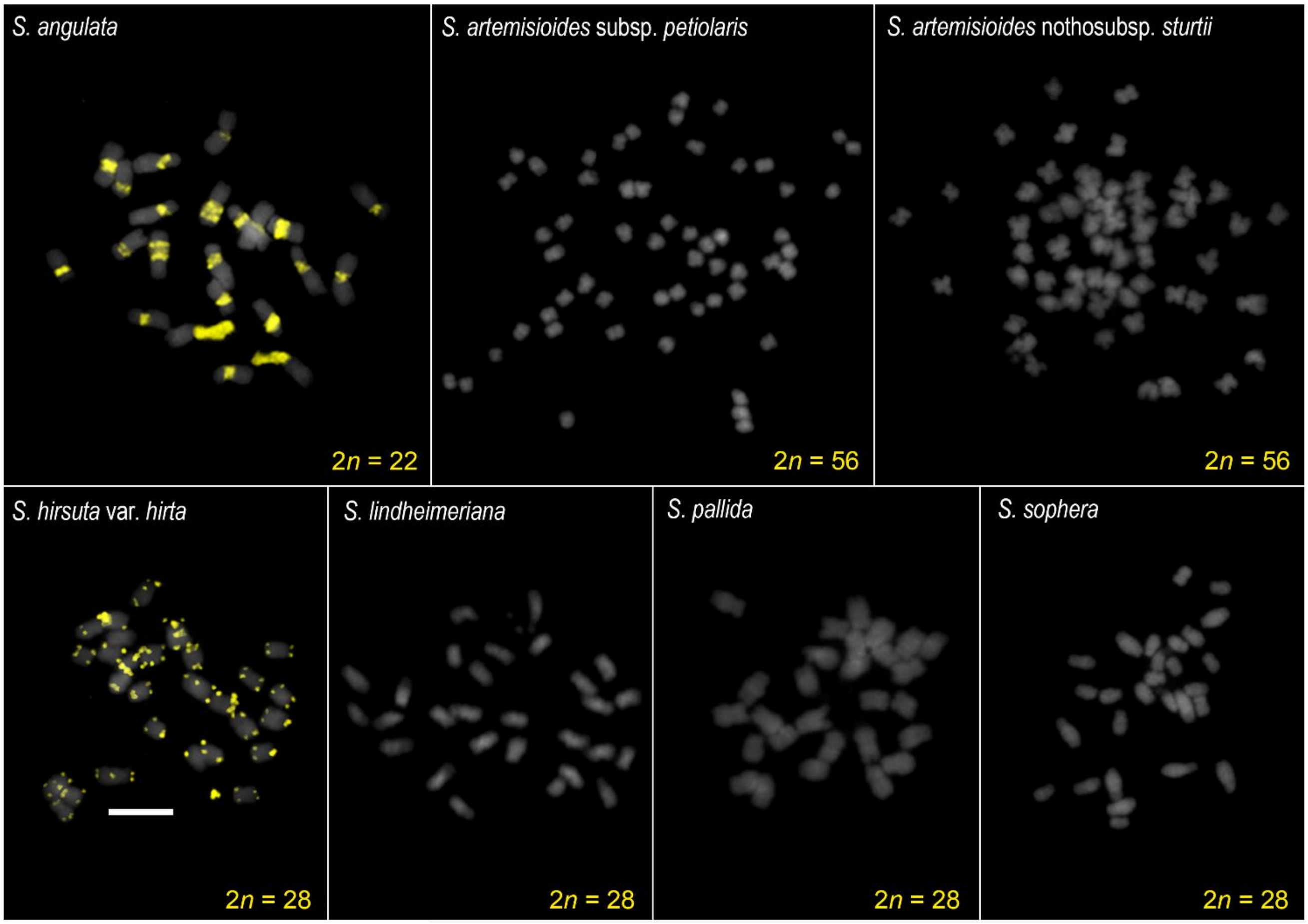

Figure 2S. Distribution of StoTR03_178 in metaphase spreads in seven *Senna* species. StoTR03_178 signal is indicated in yellow. Scale bar = 5 µm.

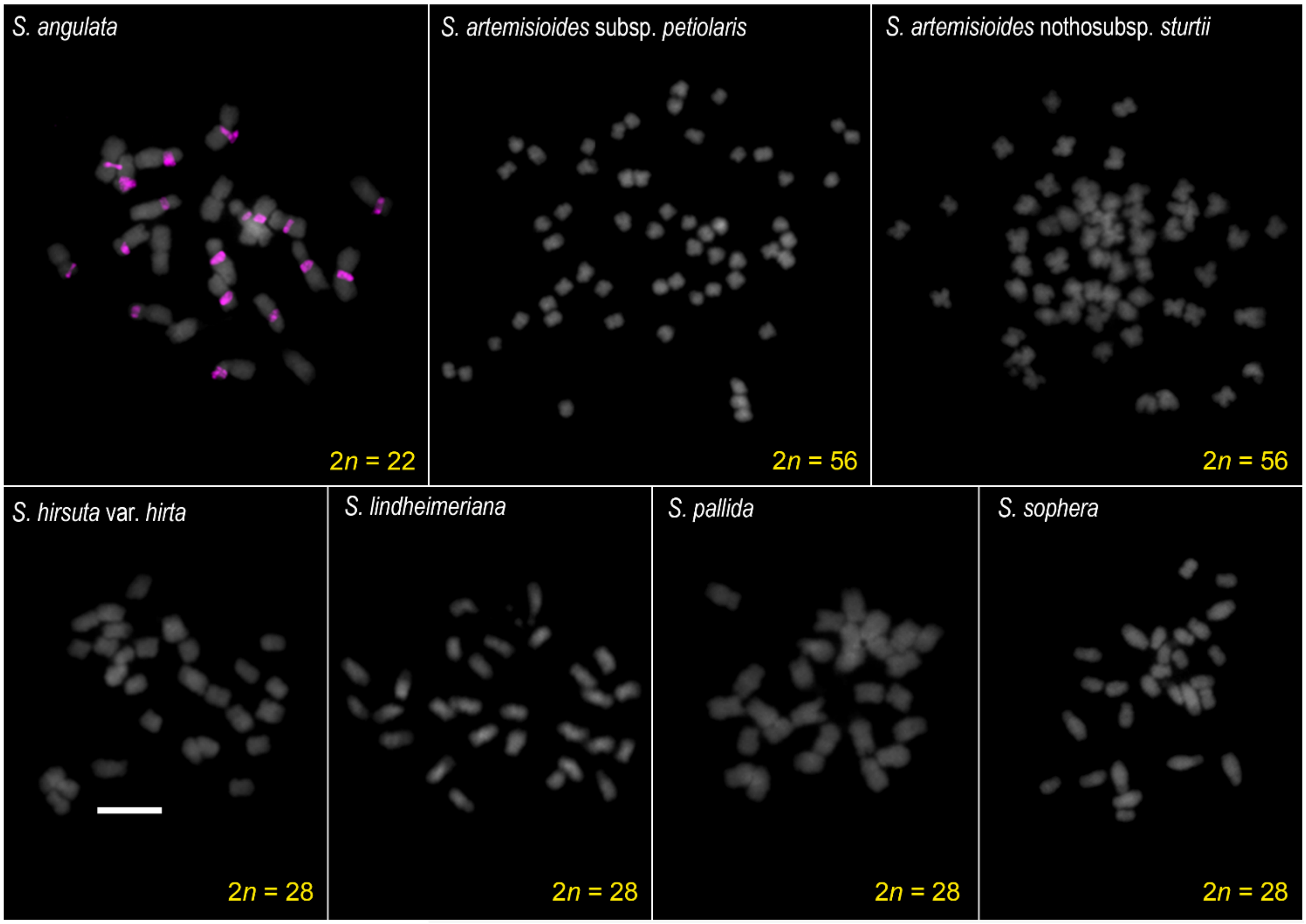

Figure 3S. Distribution of StoTR05_180 in metaphase spreads in seven *Senna* species. StoTR05_180 signals are labeled pink. Scale bar = 5 µm

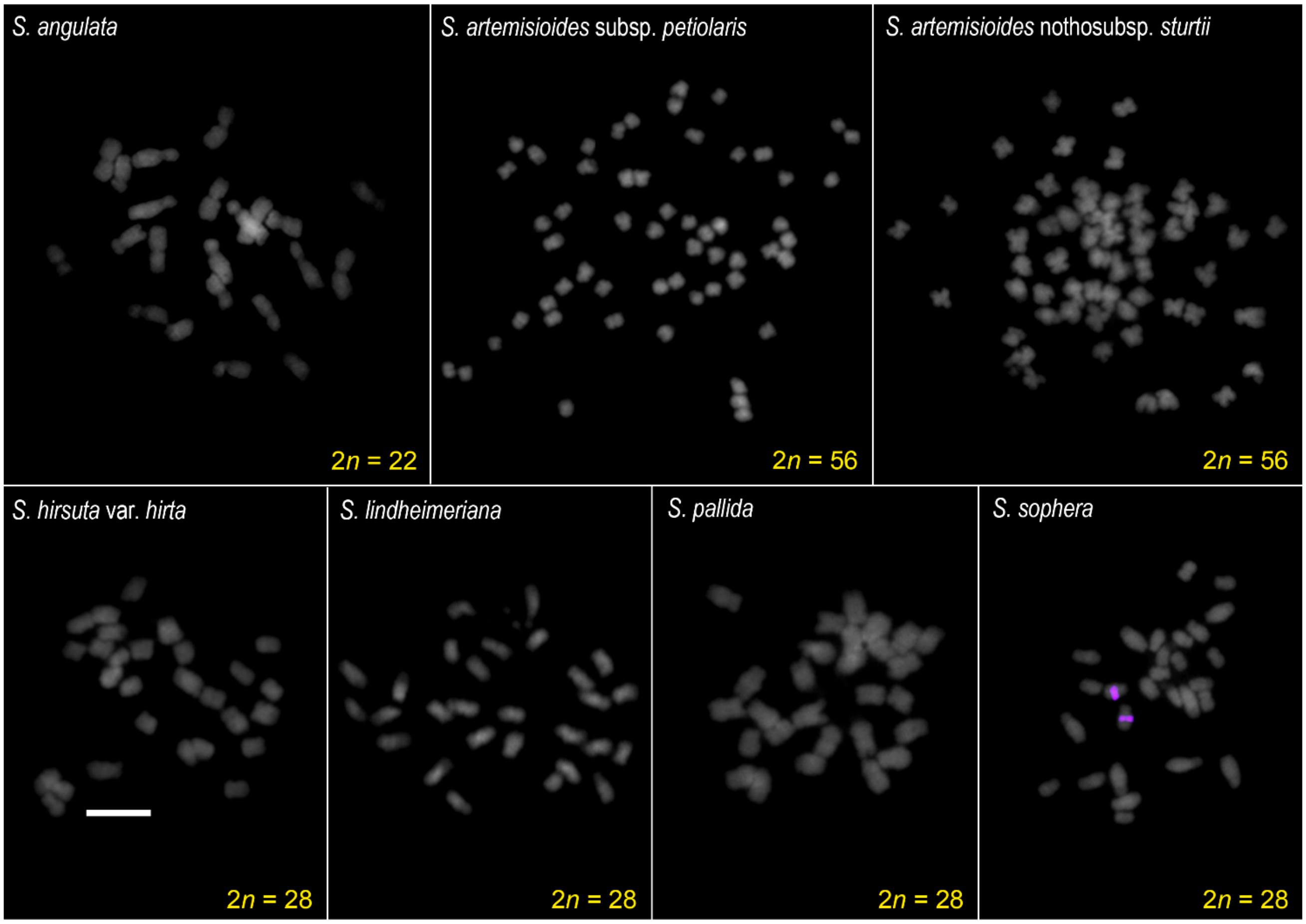

Figure 4S. Distribution of StoIGS_463 in metaphase spreads in seven *Senna* species. The StoIGS_463 signal is labeled purple. Scale bar = 5 µm

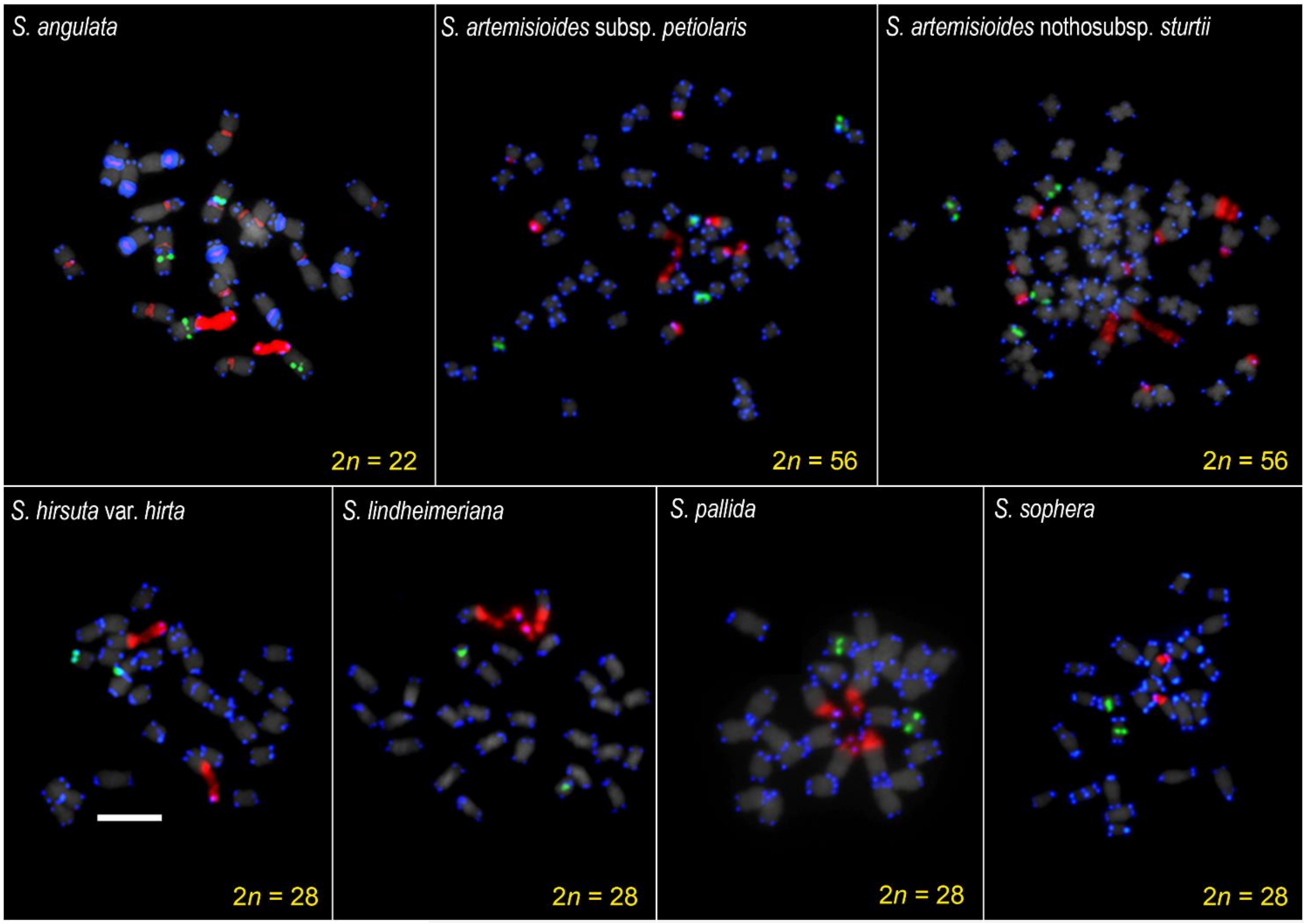

Figure 5S. Distribution of Sto_5S, Sto_45S, and Sto_Tel repeat probes in the metaphase spreads in seven *Senna* species. Sto_5S, Sto_45S, and Sto_Tel repeat signals are labeled green, red, and blue, respectively. Scale bar = 5 µm

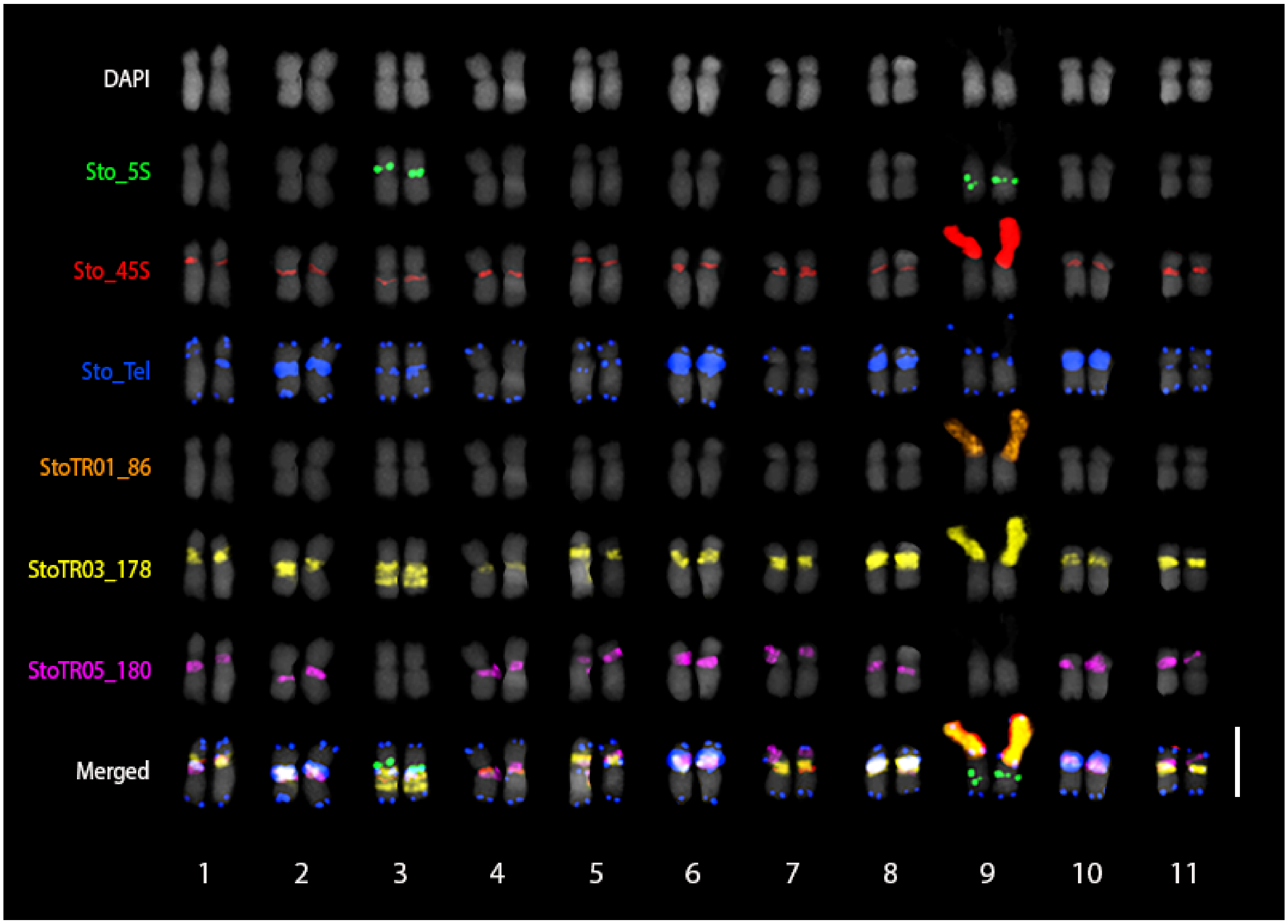

Figure 6S. FISH karyogram showing TR probe distribution in *S. angulata*. Scale bar = 5 μm

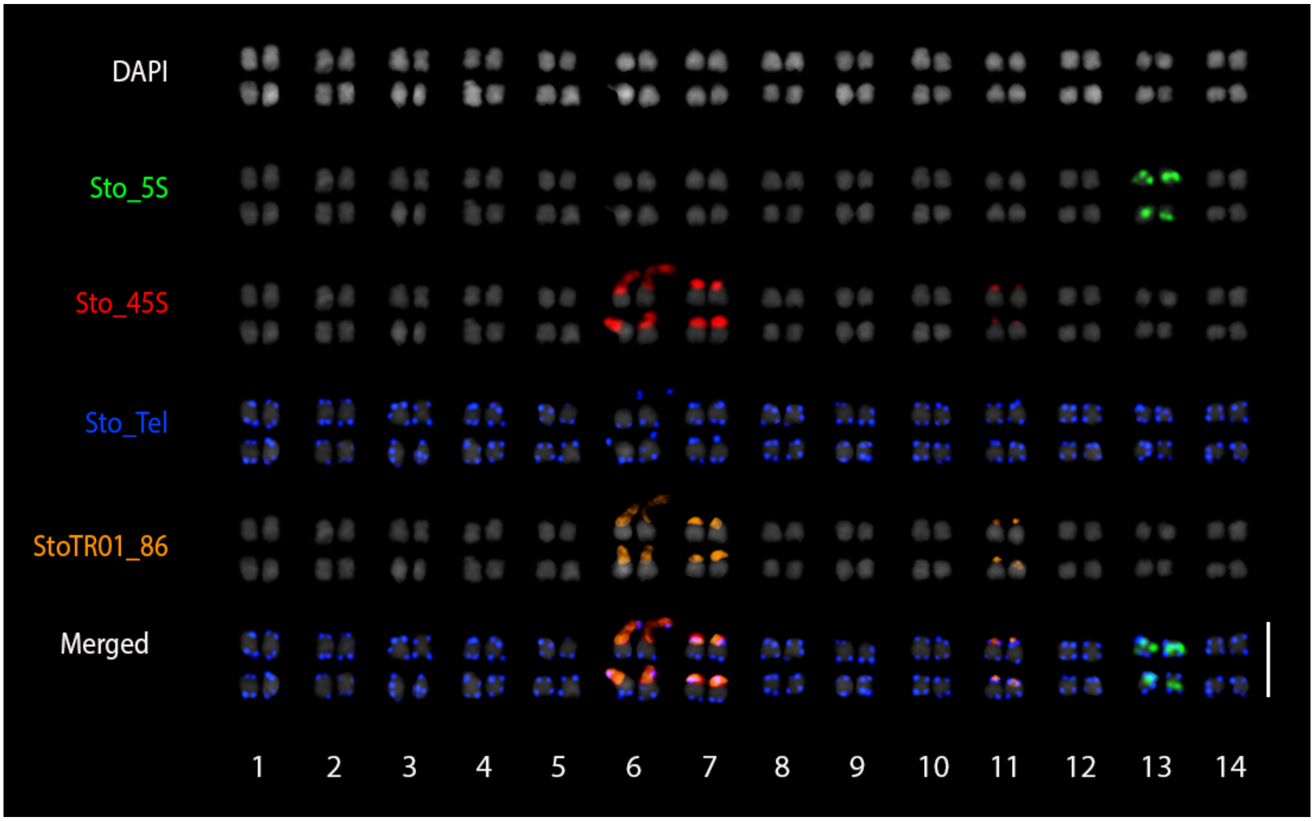

Figure 7S. FISH karyogram showing TR probe distribution in *S. artemisioides* subsp. *petiolaris*. Scale bar = 5 µm

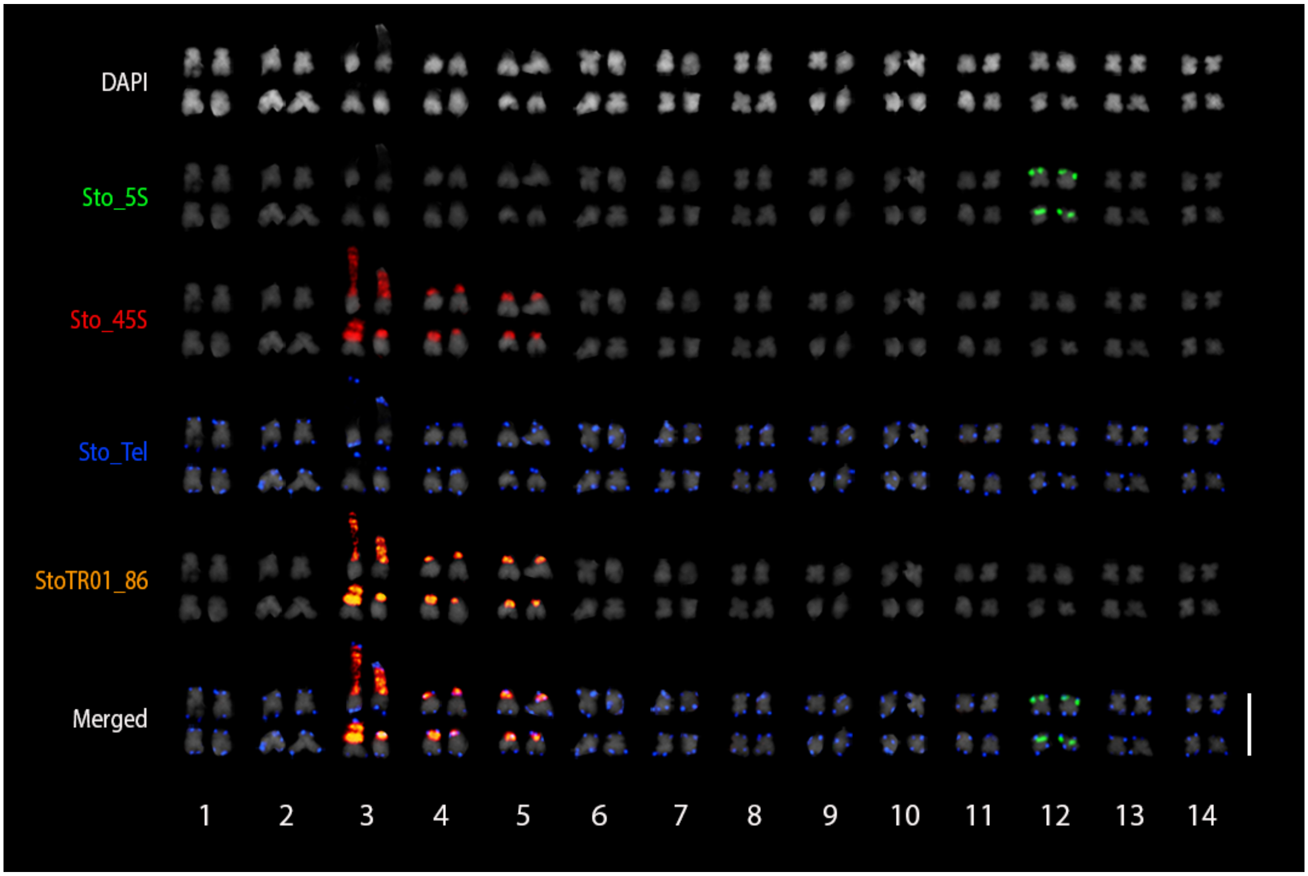

Figure 8S. FISH karyogram showing TR probe distribution in *S. artemisioides* nothosubsp. *sturtii*. Scale bar = 5 µm

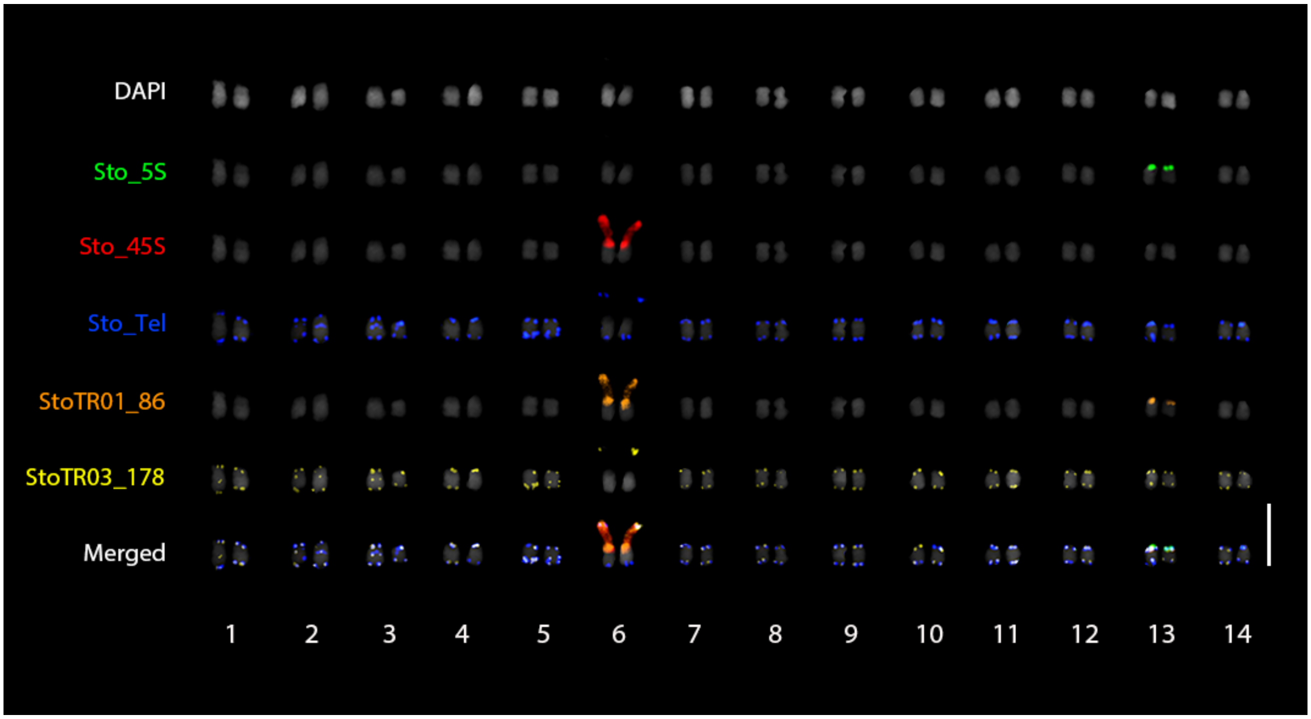

Figure 9S. FISH karyogram showing TR probe distribution in *S. hirsuta* var. *hirta*. Scale bar = 5 µm

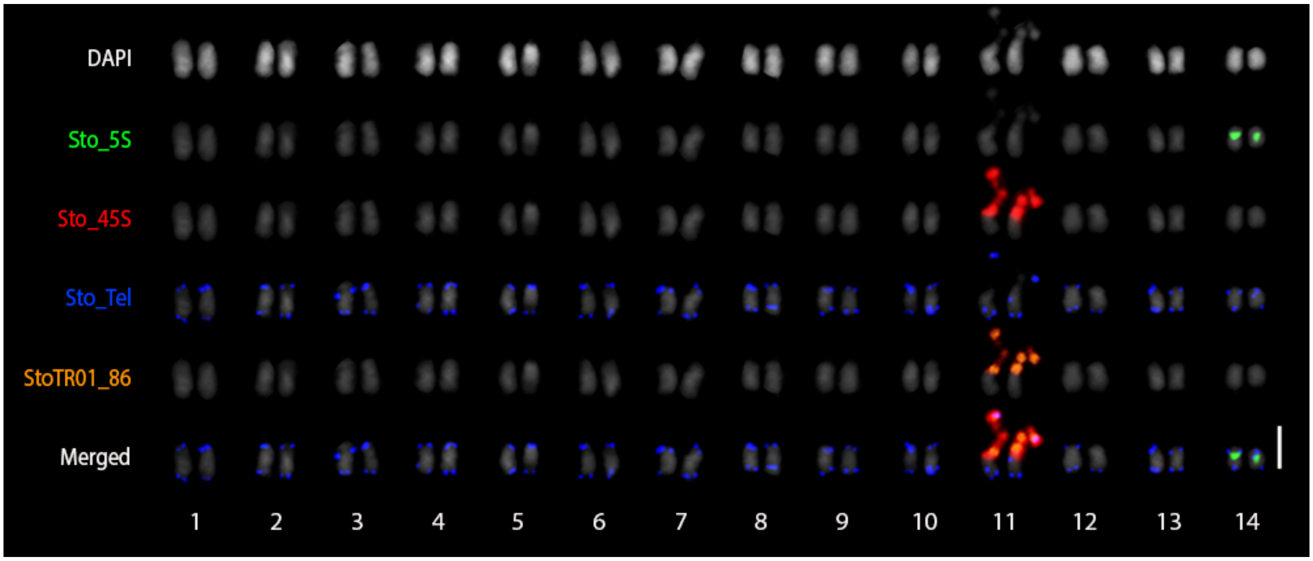

Figure 10S. FISH karyogram showing TR probe distribution in *S. lindheimeriana* using the TRs probes. Scale bar = 5 µm

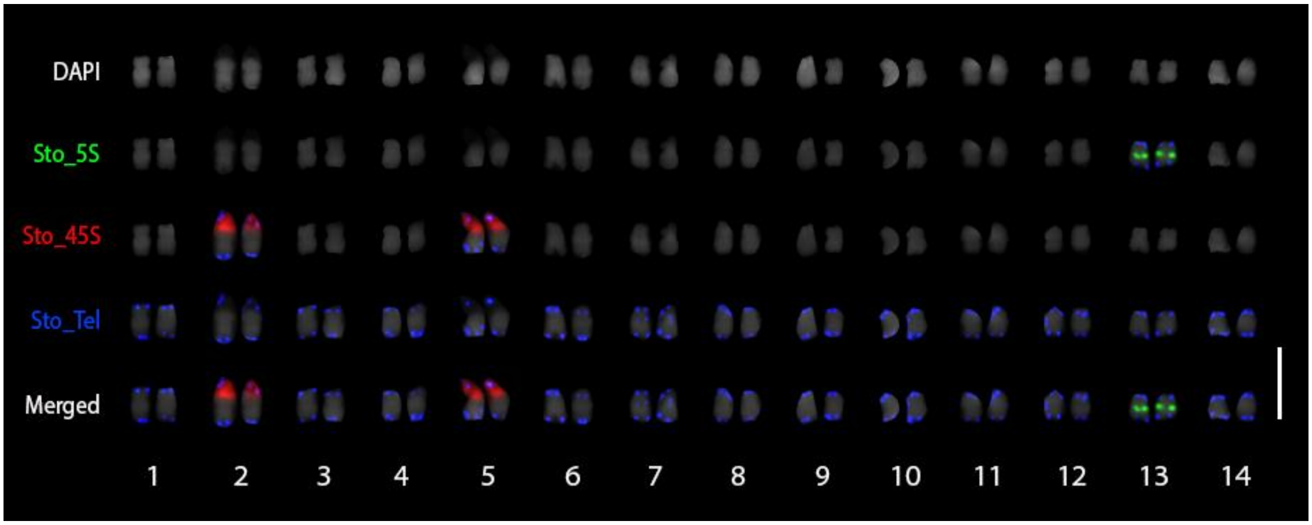

Figure 11S. FISH karyogram showing TR probe distribution in *S. pallida*. Scale bar = 5 µm

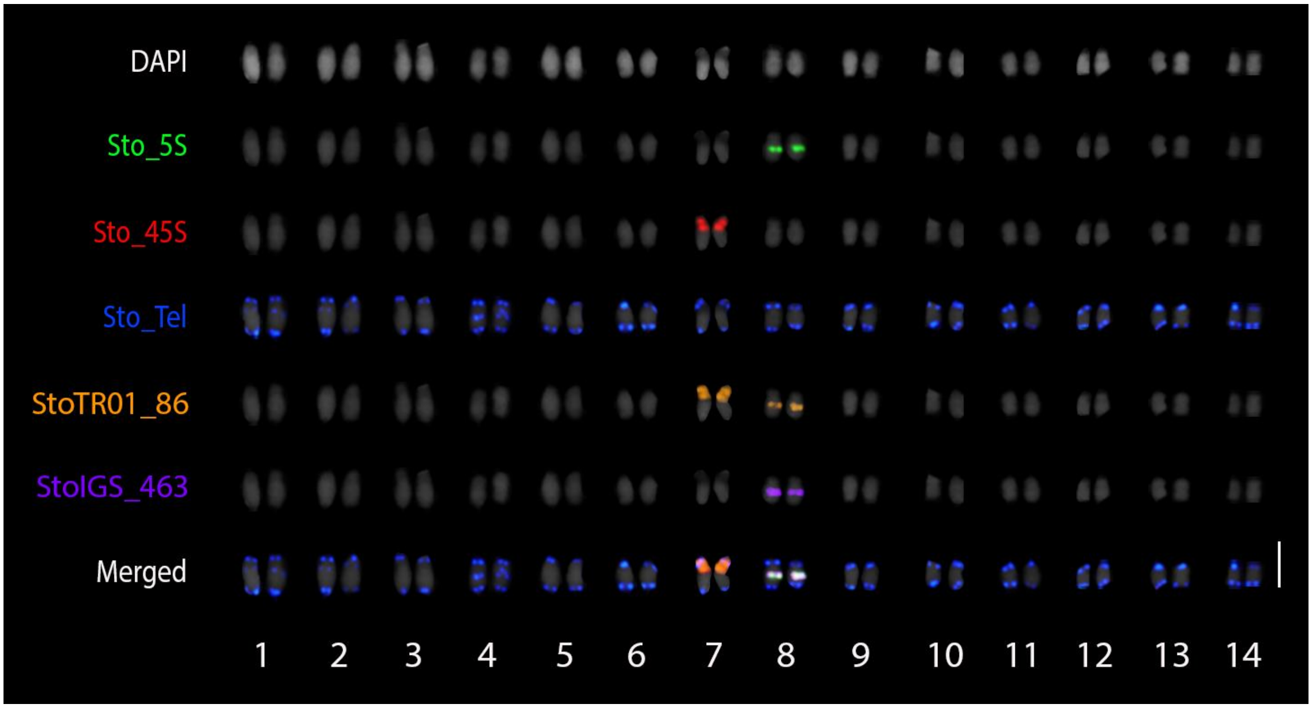

Figure 12S. FISH karyogram showing TR probe distribution in *S. sophera*. Scale bar = 5 µm
